# Supplementary material for: Satisfaction of Online University Education during the COVID-19 Pandemic
Source: Healthcare (Basel). 2023 May 14;11(10):1421. doi: 10.3390/healthcare11101421 (PMC10218284; doi:10.3390/healthcare11101421)
Supplement: Supplementary file 1 [file healthcare-11-01421-s001.zip › healthcare-2309887-supplementary.pdf]

Satisfacción de los estudiantes del Grado en Enfermería (Spanish version)

Estimado participante,

En nombre de los investigadores de este estudio, nos gustaría pedir su colaboración para llevar a cabo el mismo.

El objetivo de la investigación es conocer la satisfacción del alumnado del Grado de Enfermería de la UJA respecto a la docencia online recibida durante la pandemia por COVID-19.

La investigación la llevaremos a cabo con el alumnado de todos los cursos del Grado de Enfermería de la Universidad de Jaén, los cuales, además, acepten participar en el presente estudio, mediante la firma del consentimiento informado.

La investigación consiste en la administración de un cuestionario, donde buscamos que nos expongas tu opinión sobre la formación online recibida desde marzo de 2020 hasta la actualidad.

La información obtenida será tratada de manera agregada y anónima. Puedes revocar tu participación cuando lo desees sin necesidad de alegar ningún motivo justificado.

Para cualquier duda sobre el proyecto o para rechazar participar, por favor contactar con la profesora Lucía Ortega Donaire, a través del correo lortega@ujaen.es

Indica tu sexo

- Hombre
- Mujer
- Otro (indicar cual):

Indica tu edad

____

Indica el curso académico que cursas actualmente (si te encuentras en varios simultáneamente, indica el de mayor curso)

- Primer curso
- Segundo curso
- Tercer curso
- Cuarto curso

Señala tu estado civil actual:

- Soltero/a
- Casado/a
- Divorciado/a
- Vuido/a

¿Has recibido formación mediante medios digitales de manera exclusiva?

- Sí
- No

¿Cómo calificarías esta formación recibida con anterioridad a la pandemia por COVID-19?

____

Convives, en la actualidad con algún/a menor de edad

- Sí
- No

¿Qué parentesco compartes con estas personas?

- Hijo/a
- Hermano/a
- Primo/a
- Sobrino/a
- Otro (Indicar cual): ___________

Convives, en la actualidad con alguna persona con una edad superior a 65 años:

- Sí
- No

¿Qué parentesco compartes con estas personas?

- Abuelo/a
- Madre/padre

- Suegro/a
- Otro (Indicar cual):________

A continuación, vamos a exponer a una serie de preguntas relacionadas con la formación que has recibido durante el Grado en Enfermería.
Para responder debes indicar el grado de acuerdo con las afirmaciones que a continuación te exponemos. Te pedimos que seas totalmente sincero/a para que de esta manera podamos conocer tu opinión respecto a la formación que has recibido.
El número "1" indica "Totalmente en desacuerdo", 2, "En desacuerdo", 3, "De acuerdo" y 4 "totalmente de acuerdo"

	1	2	3	4
El programa de las asignaturas ha sido adecuado				
Los trabajos y las prácticas de los diferentes módulos han sido valiosos para poner en marcha los conocimientos adquiridos				
Se facilitó la comprensión de las cuestiones técnicas de la plataforma en algún momento durante el curso/s académico/s				
Considero adecuada la utilización de los diferentes recursos online por parte del profesorado				
El profesorado del curso semipresencial poseía buen dominio de la materia.				
Cuando fue necesario el profesor/a dió información y explicó los contenidos presentados.				
El tutor me dio buena retroalimentación sobre las tareas a realizar.				
Considero adecuada la explicación de las normas de funcionamiento del profesorado sobre el entorno formativo				
Las recomendaciones públicas o privadas sobre el trabajo y la calidad de los contenidos por parte del profesorado fueron correctas				
El profesorado realizó una adecuada animación e interacción para conseguir el máximo de participación				
Se realizaron actividades para facilitar el conocimiento entre los diferentes alumnos que formábamos parte de la asignatura.				
Los diferentes contenidos que se presentan son actuales				
El volumen de información es suficiente para la formación en los diferentes contenidos presentados.				
Los contenidos han sido fácilmente comprensibles.				
La originalidad de los contenidos fue adecuada				
El interés de los contenidos desde un punto de vista teórico es adecuado.				

Considero que el interés de los contenidos desde un punto de vista práctico es adecuado.				
Considero de los contenidos son agradables				
La relación entre los objetivos y los contenidos ofrecidos era adecuada.				
La relación entre la temporalización y los contenidos ofrecidos fue apropiada.				
Considero adecuada la calidad tanto científica como didáctica-educativa de los contenidos abordados.				
La comunicación con el profesorado me ha resultado fácil mediante las herramientas propuestas: Correo, foro, chat				
Me ha resultado fácil la comunicación online (en las clases de docencia) con el resto de compañeros.				
La plataforma virtual, provee un entorno bueno para la comunicación con los compañeros.				
El funcionamiento técnico del entorno es fácil de comprender.				
Considero adecuada la plataforma porque me ha resultado fácil navegar por ella.				
La calidad estética del entorno virtual (tamaño de letra, colores, tipo de letra) la considero adecuada				
Existe adecuación entre los diferentes elementos estéticos de la plataforma (texto, imágenes, gráficos)				
Los tiempos de respuesta de la plataforma (espera para acceder a un contenido, herramientas disponibles, pruebas de evaluación) han sido adecuados.				

Satisfaction of Nursing Degree students (English version)

Dear participant,

On behalf of the investigators of this study, we would like to ask for your collaboration to carry it out.

The objective of the research is to know the satisfaction of the students of the

UJA Nursing Degree regarding the online teaching received during the COVID-19 pandemic.

The research will be carried out with the students of all the courses of the Nursing Degree of the University of Jaén, who, in addition, agree to participate in this study, by signing the informed consent.

The investigation consists of the administration of a questionnaire, where we are looking for you to tell us your opinion about the online training you received.

from March 2020 to the present.

The information obtained will be treated in an aggregated and anonymous manner. You can revoke your participation whenever you want without needing to allege any justified reason.

For any questions about the project or to refuse to participate, please contact Professor Lucía Ortega Donaire through the email lortega@ujaen.es.

Indicate your gender

- Man
- Woman
- Other (indicate which):

Indicate your age

____

Indicate the academic year you are currently studying (if you are in several simultaneously, indicate the one with the highest course)

- First course
- Second course
- Third course
- Fourth course

Indicate your current marital status:

- Single
- Married
- Divorced
- Widowed _

Have you received training through digital media exclusively?

- Yes
- No

How would you rate this training received prior to the COVID-19 pandemic?

____

You currently live with a minor

- Yes
- No

What relationship do you share with these people?

- Child
- Sibling
- Cousin
- Nephew
- Other (Indicate which one): ___________

You currently live with someone over 65 years of age:

- Yes
- No

What relationship do you share with these people?

- Grandparent
- Mother/father
- Father-in-law
- Other (Indicate which):_ _______

Next, we are going to expose a series of questions related to the training that you have received during your degree in nursing.

To respond, you must indicate your degree of agreement with the statements that we present below. We ask you to be completely honest so that, in this way, we can know your opinion regarding the training you have received.

The number "1" indicates "Strongly disagree", 2 indicates "Disagree", 3 indicates "Agree", and 4 indicates "Strongly agree".

	1	2	3	4
The program of the course has been adequate.				
The work and practices of the different modules have been valuable in putting into practice the knowledge acquired.				
The teacher-tutor of the online modules made it easier for me to understand the technical issues of the platform.				
I consider the teacher-tutor's use of the different online resources to be adequate.				
The tutor-teacher of the online modules had a good command of the subject.				
When necessary, the tutor/teacher gave information and explained the contents presented.				
The tutor/teacher gave appropriate feedback on the activities performed.				
I consider the teacher/tutor's explanation of the rules of operation of the training environment to be adequate.				
The public or private recommendations on the work and the quality of the work by the teacher/tutor were correct.				
The tutor/teacher provided adequate encouragement and stimulated participation.				
Activities were carried out to facilitate knowledge among the students who were part of the online modules.				
The different contents presented were current.				
The volume of information is sufficient for training in the different contents presented.				
The contents presented have been easy to understand.				
I think the originality of the contents offered was adequate.				
The relevance of the contents from the theoretical point of view was adequate.				
The relevance of the contents from the practical point of view was adequate.				
I consider the contents to be pleasant.				
The relationship between the objectives and the contents presented was appropriate.				
The relationship between the timing and the contents offered was appropriate.				

I consider the scientific and didactic-educational quality of the contents addressed to be adequate.				
Communication with the teachers-tutors has been easy for me through the communication tools: mail, photo, virtual teaching platform.				
I found it easy to communicate online with the rest of my classmates in my environment.				
The virtual spaces for informal communication among classmates have been adequate.				
The technical operation of the virtual environment is easy to understand.				
I consider the online teaching platform adequate because I found it easy to navigate.				
The aesthetic quality of the environment (size, font, colors...) was adequate.				
There is adequacy between the different aesthetic elements of the platform (text, images, graphics).				
The response times of the platform (waiting to access a link, access to the different tools) were adequate.				
